# Humoral immune response to polyvalent pneumococcal vaccine in healthy participants receiving efgartigimod: a randomized, open-label, placebo-controlled, parallel-group phase 1 trial

**DOI:** 10.3389/fimmu.2026.1799480

**Published:** 2026-05-07

**Authors:** Antoine E. Azar, John W. Sleasman, Sophie Steeland, Fien M. Verhamme, Kevin L. Winthrop

**Affiliations:** 1 Division of Allergy and Clinical Immunology, Johns Hopkins University, Baltimore, MD, United States; 2 Division of Allergy and Immunology, Duke University School of Medicine, Durham, NC, United States; 3 argenx, Ghent, Belgium; 4 Division of Infectious Diseases, Oregon Health and Science University, Portland, OR, United States

**Keywords:** antibody, B cell, efgartigimod, immune response, immunoglobulin G, neonatal Fc receptor, vaccine

## Abstract

**Background/objective:**

Efgartigimod, a human immunoglobulin G1 (IgG1) antibody Fc fragment, reduces IgG levels through neonatal Fc receptor (FcRn) blockade. Preliminary observational data suggest that efgartigimod does not impair vaccine-induced T cell–dependent antibody responses. The objective of this study was to further evaluate the effect of efgartigimod on T cell–independent humoral immune response to immunization with a 23-valent polysaccharide pneumococcal vaccine (PPSV23).

**Methods:**

In this Phase 1 open-label study, healthy adults (N=37) were randomized 1:1:1 into 3 cohorts: EFG-1 (n=12), EFG-2 (n=12), and placebo (n=13). Four weekly efgartigimod (10 mg/kg intravenously) or placebo infusions were administered with a 6-week follow-up. PPSV23 was administered immediately before (EFG-1/placebo) or 2 weeks after (EFG-2) the last efgartigimod or placebo infusion. The primary endpoint was change in pneumococcal capsular polysaccharide titers after PPSV23 vaccination. Additional endpoints included opsonizing antibody titers, antigen-specific B cell responses, and safety.

**Results:**

Although variability in antibody response was observed for individual participants and serotypes, an increase in IgG levels was observed for all 23 pneumococcal serotypes after vaccination, irrespective of efgartigimod timing. Normal response to vaccination (≥2-fold increase and >1.3 mg/L for ≥70% of serotypes) was observed in 90.0% (9 of 10) of the EFG-1 group, 54.5% (6 of 11) of the EFG-2 group, and 33.3% (4 of 12) of the placebo group. Efgartigimod did not impair production of opsonizing antibodies or antigen-specific plasma B cells. No serious/severe adverse events occurred.

**Conclusions:**

Results of this exploratory study suggest that efgartigimod has no apparent effect on T cell–independent humoral immune responses to PPSV23 vaccine in healthy adults. Participants in all groups were able to mount antigen-specific IgG responses to vaccination, even when vaccination occurred at the time of maximal reduction in total IgG.

**Clinical Trial Registration:**

https://clinicaltrials.gov/study/NCT05163834, identifier NCT05163834.

## 1 Introduction

Autoimmune diseases encompass a group of chronic, disabling conditions that collectively affect up to 8% of the global population (1, 2). The incidence and prevalence of autoimmune diseases have increased over the past few decades (3) and represent an expanding contributor to global mortality (4), morbidity (4), and economic burden (5). Pathogenic autoantibodies, primarily of the immunoglobulin G (IgG) isotypes, are directly or indirectly implicated in many autoimmune diseases, including myasthenia gravis (MG), primary immune thrombocytopenia (ITP), chronic inflammatory demyelinating polyradiculoneuropathy (CIDP), thyroid eye disease, and primary Sjogren’s disease (6–9). Routine management of autoantibody-mediated diseases often involves systemic immunosuppression using corticosteroids, nonsteroidal immunosuppressants (eg, mycophenolate mofetil, azathioprine), B cell–depleting therapies, or some combination thereof (1, 10). While these therapies are effective in managing autoantibody-mediated autoimmune diseases for many patients (9, 10), they are also variably associated with increased risks of malignancy and infection, potentially as a consequence of nonselective systemic immunosuppression (11–17).

Although systemic immunosuppression and underlying immune dysregulation may increase the risk of infections in patients with autoimmune diseases, some of this risk can be mitigated by vaccination (18). The US Advisory Committee on Immunization Practices recommends administration of certain vaccines, such as shingles and pneumococcal vaccines, outside the routine age-based recommendations in immunocompromised patients (19–22). However, suppression of the adaptive immune system by systemic immunosuppressive agents can decrease the ability to initiate and maintain immune responses needed for effective vaccination (17, 18, 23–26). Accordingly, there remains an unmet need for more selective treatment strategies that target the pathophysiology of IgG-mediated autoimmune diseases while minimizing the effects on cellular and humoral immune responses to infections and vaccines.

The neonatal fragment crystallizable (Fc) receptor (FcRn) is a key driver of IgG autoantibody recycling and preservation (27, 28). FcRn-mediated recycling results in IgG having the longest half-life and being the most abundant of all immunoglobulins, thereby contributing to the pathogenesis of IgG autoantibody–mediated diseases (28–31). Efgartigimod is a human IgG1 antibody Fc fragment engineered for high affinity to FcRn that selectively reduces IgG levels by blocking FcRn-mediated IgG recycling without impacting antibody production (27, 30, 32). Efgartigimod reduces all IgG isotypes without affecting levels of other immunoglobulins (ie, IgA, IgD, IgE, IgM), albumin concentration, and the generation of a humoral or cellular immune response (27, 30, 32–36). Efgartigimod has received regulatory approval in multiple regions for patients with generalized MG (gMG) (37–41), was approved for ITP in Japan (42) and for CIDP in the United States and Japan (38, 43), and is being investigated in multiple other IgG autoantibody–mediated diseases (32, 44, 45). As efgartigimod is the first FcRn inhibitor to receive regulatory approval (40), clarifying whether it impacts effective immune responses to vaccines is an area of clinical interest. Preclinical studies and *post hoc* analyses of clinical studies have suggested that efgartigimod does not impair generation of IgG responses to T cell–dependent or –independent vaccines (27, 35, 36, 46, 47). However, this has not yet been evaluated in a randomized, placebo-controlled trial.

To further evaluate whether efgartigimod affects immunogenicity to T cell–independent vaccines and provide additional clarity on administering vaccines to patients receiving efgartigimod, we conducted a placebo-controlled study evaluating humoral immune responses to the 23-valent polyvalent pneumococcal polysaccharide vaccine (PPSV23; PNEUMOVAX 23). PPSV23 contains a mixture of purified capsular polysaccharides from 23 different *Streptococcus pneumoniae* types (48) and elicits an IgG response independent of T cell help, resulting in a more simplified pathway to IgG secretion and a faster protective response (49–51). Healthy volunteers were randomized 1:1:1 to receive PPSV23 immediately before (EFG-1 and placebo) or 2 weeks after (EFG-2) the last of 4 once-weekly infusions of intravenous (IV) efgartigimod or placebo.

## 2 Methods

### 2.1 Study design and participants

This was a Phase 1, exploratory, randomized, placebo-controlled, open-label, parallel-group trial (ClinicalTrials.gov identifier, NCT05163834; WHO ICTRP identifier, NL-OMON50337). The trial was conducted in accordance with Good Clinical Practice guidelines and conformed to the ethical principles of the Declaration of Helsinki and relevant country-specific laws, with approval by the Independent Ethics Committee of the Stichting Beoordeling Ethiek Biomedisch Onderzoek (Assen, the Netherlands; Central Committee on Research Involving Human Subjects code NL79381.056.21; approval date, November 12, 2021). Written informed consent was obtained from all trial participants prior to enrollment, which began on November 17, 2021.

Healthy adults with a body mass index (BMI) between 18 kg/m^2^ and 30 kg/m^2^ were recruited for this study. Medical evaluation was used to determine whether a participant was considered healthy. This evaluation comprised medical history, physical examination, laboratory tests (including hematology, clinical chemistry, routine urinalysis, and specialty tests [apolipoprotein B, fibrinogen, lipoprotein A]), and cardiac monitoring (including heart rate, atrioventricular nodal delay [PR], duration of ventricular depolarization [QRS], total duration of ventricular depolarization [QT], and intervals). The findings of the medical evaluation were interpreted by the investigator, and if results were not deemed to be clinically significant, participants were eligible to enroll in the study. Participants were excluded if they had total IgG ≤6 g/L at screening, had used any systemic immunosuppressant agent within 6 months or any systemic steroid within 3 months prior to initiating study treatment, had a pneumococcal infection within the past 5 years, had received a live or live-attenuated vaccine <4 weeks before screening, or had received a pneumococcal vaccine within the past 10 years. A full list of inclusion and exclusion criteria can be found at ClinicalTrials.gov.

**Figure 1:**
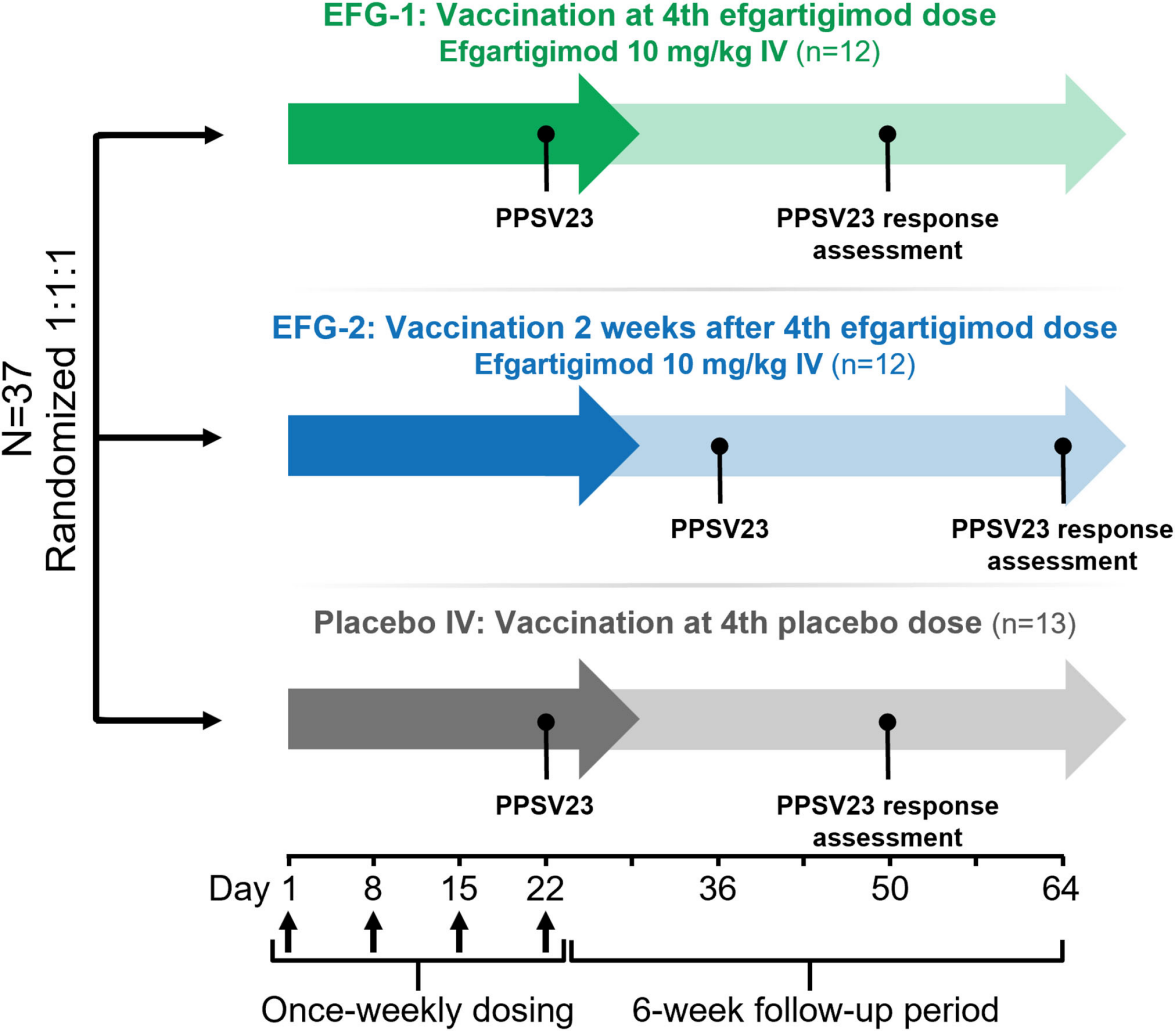
Study design. Thirty-seven healthy volunteers were randomized 1:1:1 to 1 of 3 treatment groups. Participants received either efgartigimod 10 mg/kg or matching placebo IV on Days 1, 8, 15, and 22, followed by a 6-week follow-up period. PPSV23 was administered on Day 22 in the EFG-1 and placebo groups and on Day 36 in the EFG-2 group. EFG, efgartigimod; IV, intravenously; PPSV23, 23-valent polyvalent pneumococcal polysaccharide vaccine.

The study consisted of a ≤3-week screening period, a 3-week study treatment period, and a 6-week follow-up period. Patients were centrally randomized 1:1:1 to receive 1 cycle (ie, 4 once-weekly IV infusions) of efgartigimod 10 mg/kg plus PPSV23 (Merck Sharp & Dohme Corp.; batch number 1036859) either immediately before (EFG-1 arm) or 2 weeks after (EFG-2 arm) the fourth infusion, or 4 weekly IV infusions of placebo plus PPSV23 immediately before the fourth infusion (placebo arm; **Figure 1**). PPSV23, containing polysaccharide antigens corresponding to pneumococcal serotypes 1, 2, 3, 4, 5, 6B, 7F, 8, 9N, 9V, 10A, 11A, 12F, 14, 15B, 17F, 18C, 19A, 19F, 20, 22F, 23F, and 33F (25 µg of each), was administered as a 0.5-mL intramuscular injection (48).

### 2.2 Assessments

The primary endpoint was the variation in immune response as assessed by comparing pneumococcal capsular polysaccharide titers before and after administration of PPSV23. Serum pneumococcal capsular polysaccharide titers were assessed on Day 1 (baseline), on Day 22 (end of treatment period), and on Days 36, 50, and 64 during the follow-up period (or, in those who discontinued early, at safety follow-up 56 ± 3 days after their last study treatment). Titers were measured in a specialty laboratory (Quest Nichols Institute, Valencia, California, USA) by a validated bead-based multi-analyte immunodetection method using Luminex MagPlex Microspheres (a lower limit threshold of 0.3 µg/mL was used for all serotypes; upper limit thresholds are reported in **Supplementary Table 1**) (52). Secondary endpoints included the incidence and severity of adverse events (AEs), which were reported by the participants.

To support the primary endpoint, the number of participants who achieved a normal response at 4 weeks postvaccination (Day 50 for the EFG-1 and placebo groups and Day 64 for the EFG-2 group) was assessed. Normal response was defined by simultaneous achievement of both a ≥2-fold increase and absolute value >1.3 mg/L in the antibody titers for ≥70% of serotypes (53–56). Additional *post hoc* analyses assessed the number of participants in each study arm who achieved either individual response criterion (ie, postvaccination antibody level >1.3 mg/L or ≥2-fold increase in antibody concentration) for ≥70% of serotypes.

Exploratory endpoints included the variation in the immune response as assessed by comparing functional antibody titers and antigen-specific B cell response before and after administration of PPSV23. To assess the functionality of anti–pneumococcal capsular polysaccharide antibodies, the opsonophagocytic capacity of antibodies to the 8 most prevalent pneumococcal serotypes (1, 4, 5, 6B, 7F, 14, 19A, and 23F) was measured 4 weeks postvaccination using a validated multiplex opsonophagocytic assay that reproduces the *in vivo* protective mechanism of these antibodies (57, 58), specifically their ability to opsonize pneumococcal bacteria (World Health Organization Pneumococcal Serology Reference Laboratory, London, UK). Since PPSV23 is a T cell–independent vaccine, B cell–dependent immune response was assessed in peripheral blood mononuclear cells (PBMCs) using a commercially available single-color enzymatic B cell ELISpot assay (Human IgG Single-Color ELISPOT, Cellular Technology Limited, catalog number hIgG-SCE). The test antigen used for coating the wells contained 20 polysaccharides from *Streptococcus pneumoniae* (supplied by American Type Culture Collection) combined into a single antigen pool. Changes in total IgG levels over time and from baseline were also evaluated as an exploratory endpoint using a validated immunoturbidimetric assay (Tina-quant IgG Gen.2; Roche Diagnostics GmbH, Mannheim, Germany) (59).

### 2.3 Statistical analysis

Randomized participants who received ≥1 dose of efgartigimod or placebo were included in the safety analysis set. Participants in the safety analysis set who received PPSV23 were included in the pharmacodynamic (PD) analysis set, which was the analysis population for the primary endpoint and all other PD endpoints.

This was an exploratory study; no sample size calculations were performed, and no statistical hypotheses were tested. The primary endpoint was analyzed using descriptive statistics. Pneumococcal capsular polysaccharide titers (geometric mean of absolute value, geometric mean fold rise [GMFR] from prevaccination, and percent change from prevaccination) were summarized by serotype at relevant time points. Geometric mean opsonization titers were assessed for the 8 serotypes in PPSV23, and the proportion of patients with serotype titers exceeding the lower limit of quantification (LLOQ) was determined for each group. Change in total IgG was analyzed using descriptive statistics (mean absolute value, change from baseline, and percent change from baseline). Categorical exploratory vaccination response data were summarized using participant counts and percentages. All AE summaries include only treatment-emergent AEs (TEAEs). The number of events, frequency, and percentage of TEAEs were presented by System Organ Class and Preferred Term according to the Medical Dictionary for Regulatory Activities Version 24.1. TEAEs were also summarized by severity and relationship to efgartigimod or placebo.

## 3 Results

### 3.1 Participants

Participant disposition is summarized in **Supplementary Figure 1**. Between November 2021 and March 2022, 82 individuals were screened. Of those screened, 37 enrolled in the study and were randomized to EFG-1 (n=12), EFG-2 (n=12), or placebo (n=13). Thirty-six total participants (12 in each group) received ≥1 infusion of efgartigimod or placebo and were included in the safety analysis set; of these participants, 34 received all 4 infusions of efgartigimod (EFG-1, n=10; EFG-2, n=12) or placebo (n=12). Thirty-three participants (EFG-1, n=10 [83.3%]; EFG-2, n=11 [91.7%]; placebo, n=12 [100%]) received PPSV23 and were included in the PD analysis set. Four patients withdrew from the study (EFG-1, n=2 [17%]; EFG-2, n=1 [8%]; placebo, n=1 [8%]), of whom 3 (2 from the EFG-1 arm and 1 from the EFG-2 arm) withdrew due to AEs. One participant who was randomized to placebo withdrew before dosing due to vomiting, after which an additional participant was randomized to placebo.

Baseline demographics were generally well balanced among the groups (**Table 1**). Notably, participants in the placebo group were older (mean age, 61 years) and had a higher mean BMI (25.7 kg/m^2^) than the other 2 groups, and the EFG-1 group had fewer male participants (n=2 [16.7%]) than the EFG-2 and placebo groups (both n=6 [50%]). These differences in age and sex between groups are due to the lack of age and sex stratification in the randomization process with a small sample size and broad age range.

**Table 1:** Baseline demographic and key laboratory parameters (safety analysis set).

Category	EFG-1 (n=12)	EFG-2 (n=12)	Placebo (n=12)
Age y^a^			
Mean (SD) Median	46 (22) 45	47 (20) 49	61 (19) 66
BMI kg/m^2^, mean (SD)^a^	24.9 (2.0)	23.9 (3.0)	25.7 (2.5)
Sex n (%).			
Male Female	2 (16.7) 10 (83.3)	6 (50.0) 6 (50.0)	6 (50.0) 6 (50.0)
Race n (%)			
White White + Asian Black or African American + White	12 (100) 0 0	11 (91.7) 0 1 (8.3)	11 (91.7) 1 (8.3) 0
Ethnicity n (%)			
Not Hispanic or Latino	12 (100)	12 (100)	12 (100)
Baseline IgG mg/dL, mean (SD)^b^	994 (255)	1141 (210)	1060 (205)

^a^ Age and BMI at screening are shown. ^b^Pharmacodynamic population (EFG-1, n=10; EFG-2, n=11; placebo, n=12); baseline is defined as the last observation recorded before the first study drug administration on Day 1. BMI, body mass index; EFG, efgartigimod; IgG, immunoglobulin G.

### 3.2 Pharmacodynamic assessments

**Figure 2:**
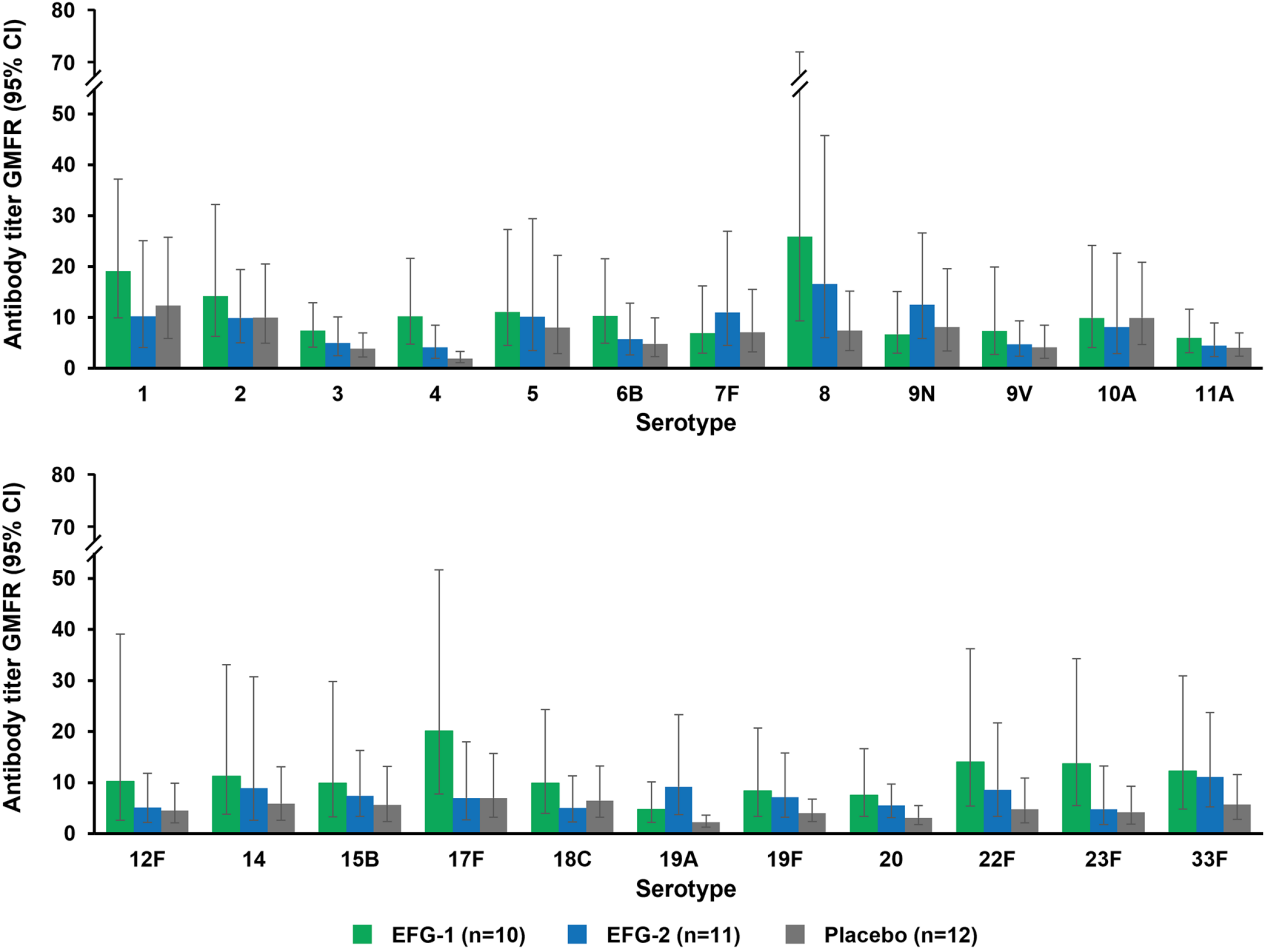
Geometric mean fold rise from prevaccination to postvaccination in pneumococcal capsular polysaccharide titers in the EFG-1, EFG-2, and placebo groups. Postvaccination titers were obtained 4 weeks after receiving PPSV23 (on Day 50 for the EFG-1 and placebo groups and Day 64 for the EFG-2 group). GMFRs for all *Streptococcus pneumoniae* serotypes contained within PPSV23 are distributed across the top and bottom graphs in each panel for legibility. EFG, efgartigimod; GMFR, geometric mean fold rise; PPSV23, 23-valent polyvalent pneumococcal polysaccharide vaccine.

Considerable variability in antibody response was observed among individual participants and serotypes. Nevertheless, an overall increase across all groups in IgG levels was observed from prevaccination to postvaccination for all 23 pneumococcal serotypes, as evidenced by GMFR (**Figure 2**) and mean percent change in pneumococcal capsular polysaccharide titers (**Supplementary Figure 2**). A summary of the titers for all 23 serotypes on Day 1 and 4 weeks postvaccination is included in **Supplementary Table 2**. Baseline geometric mean titers were generally higher for most serotypes in the placebo group compared with the efgartigimod groups.

**Figure 3:**
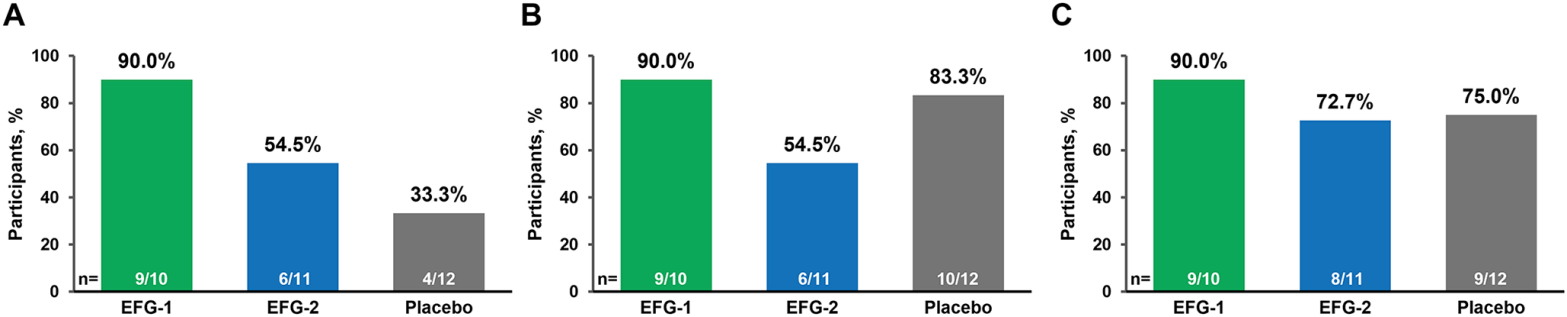
Summary of responses to pneumococcal polysaccharide vaccine (serotypes combined) 4 weeks after vaccination. Antibody concentration was measured 4 weeks after receiving PPSV23 (on Day 50 for the EFG-1 and placebo groups and Day 64 for the EFG-2 group). Panel **(A)** shows the percentages of participants with a normal response, defined as simultaneous achievement of both a ≥2-fold increase and absolute concentration >1.3 mg/L in antibody titers for ≥70% of pneumococcal serotypes (55). Panel **(B)** shows the percentage of participants who achieved antibody concentrations above >1.3 mg/L for ≥70% of pneumococcal serotypes, which is the proposed threshold for a protective titer (53–55). Panel **(C)** shows the percentage of participants with a ≥2-fold increase in antibody concentration for ≥70% of pneumococcal serotypes. EFG, efgartigimod; PPSV23, 23-valent polyvalent pneumococcal polysaccharide vaccine.

A normal response to vaccination, defined as simultaneously achieving both a ≥2-fold increase in antibody concentration and absolute concentration >1.3 mg/L in the antibody titers for ≥70% of serotypes, was observed in 90.0% (9 of 10) of the EFG-1 group, 54.5% (6 of 11) of the EFG-2 group, and 33.3% (4 of 12) of the placebo group at 4 weeks postvaccination (**Figure 3A**). In the EFG-2 group, 1 participant met both criteria for normal response in 15 of 23 (65.2%) serotypes, just missing the 70% cutoff; had this participant reached the ≥70% threshold, 63.6% of participants in the EFG-2 group would have had a normal response. Similarly, 3 participants in the placebo group met normal response criteria for 16 of 23 (69.6%) serotypes; had these participants met the ≥70% threshold, 58.3% of participants in the placebo group would have achieved a normal response. In *post hoc* analyses focused on each of the individual criteria defining a normal response, antibody levels exceeding 1.3 mg/L were observed for ≥70% of serotypes in 90.0% (9 of 10) of participants in the EFG-1 group, 54.5% (6 of 11) of participants in the EFG-2 group, and 83.3% (10 of 12) of participants in the placebo group at 4 weeks postvaccination (**Figure 3B**). A total of 90.0% (9 of 10) of participants in the EFG-1 group, 72.7% (8 of 11) in the EFG-2 group, and 75.0% (9 of 12) in the placebo group achieved a ≥2-fold increase compared with prevaccination levels in antibody concentration for ≥70% of serotypes at 4 weeks postvaccination (**Figure 3C**). The percentages of participants achieving normal response and each of the individual response criteria for each serotype are summarized in **Supplementary Table 3**.

**Figure 4:**
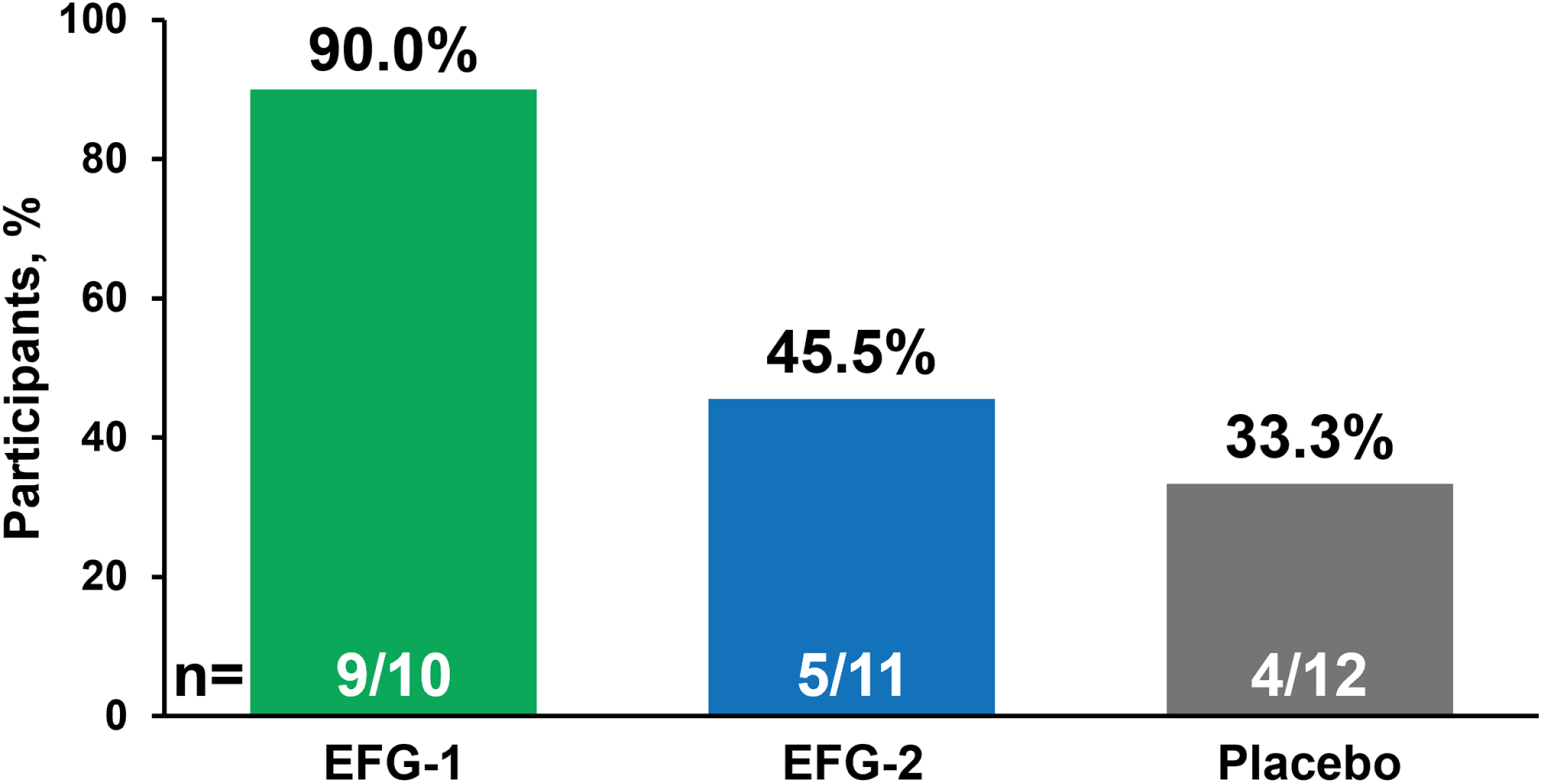
Proportion of participants within each group with opsonization titers above the LLOQ 4 weeks after vaccination with PPSV23 (Day 50 in the EFG-1 and placebo groups and Day 64 in the EFG-2 group). The assessed serotypes included 1, 4, 5, 6B, 7F, 14, 19A, and 23F. EFG, efgartigimod; LLOQ, lower limit of quantification; PPSV23, 23-valent polyvalent pneumococcal polysaccharide vaccine.

The ability of anti-pneumococcal antibodies to opsonize pneumococcal bacteria was also evaluated for the 8 most prevalent pneumococcal serotypes. At 4 weeks after vaccination, geometric mean opsonization titers increased above the LLOQ for all 8 serotypes in all study arms (**Supplementary Figure 3**). In total, 90.0% (9 of 10) of participants in the EFG-1 group, 45.5% (5 of 11) in the EFG-2 group, and 33.3% (4 of 12) in the placebo group had functional antibody titers above the LLOQ for all 8 serotypes (**Figure 4**). Opsonization titers increased after vaccination in both efgartigimod groups, but there was greater variability among participants in the EFG-2 group compared with the EFG-1 group. Opsonization titers displayed a temporal and serotype-specific pattern broadly consistent with the corresponding IgG responses. When including participants with titers above the LLOQ for 6 or more of the 8 serotypes assessed, the percentages increased to 54.5% (6 of 11) in the EFG-2 group and 58.3% (7 of 12) in the placebo group (data not shown).

**Figure 5:**
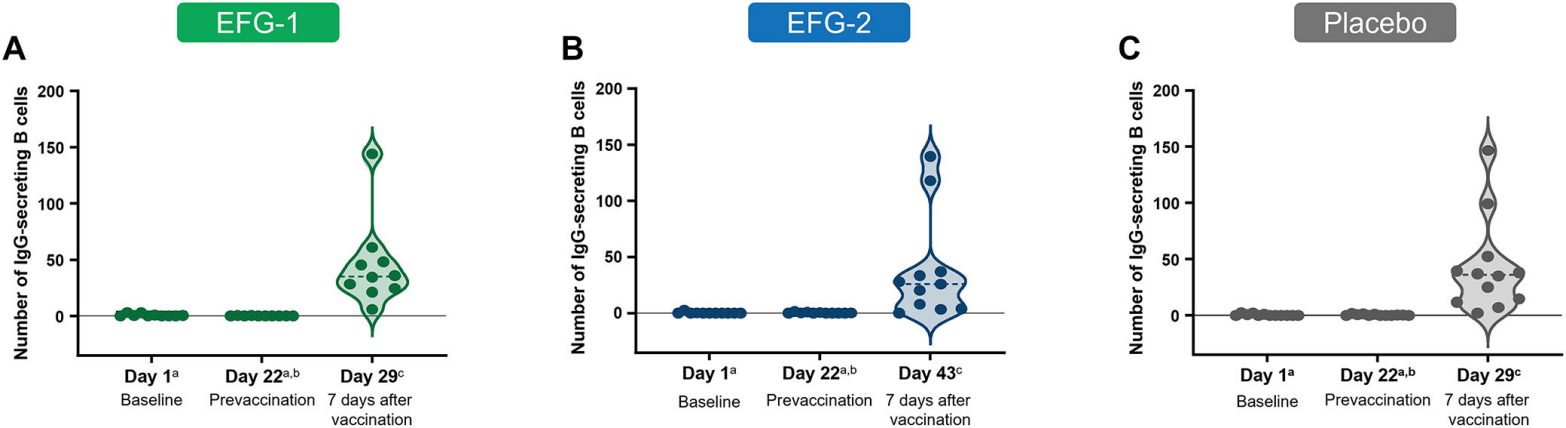
Number of antigen-specific IgG-secreting plasma B cells at baseline, prevaccination, and 7 days after vaccination in the EFG-1 [**(A)** n=10)], EFG-2 [**(B)** n=11], and placebo [**(C)** n=12] groups. The number of antigen-specific IgG-secreting plasma B cells was measured in PBMC samples using a single-color B cell ELISpot assay. Each dot represents 1 participant. ^a^Absolute values with derived number of cells <0 were set to 0. ^b^The prevaccination PBMC sampling occurred on Day 22, the same day as PPSV23 administration in the EFG-1 and placebo groups and 14 days before PPSV23 administration in the EFG-2 group on Day 36. ^c^Horizontal dashed line indicates the median. EFG, efgartigimod; ELISpot, enzyme-linked immunosorbent spot; IgG, immunoglobulin G; PBMC, peripheral blood mononuclear cells; PPSV23, 23-valent polyvalent pneumococcal polysaccharide vaccine.

The number of antigen-specific IgG-secreting plasma B cells was measured in PBMC samples collected on Day 1 (baseline), Day 22 (prevaccination), and 7 days postvaccination (Day 29 for the EFG-1 and placebo groups and Day 43 for the EFG-2 group). A total of 9 of 10 (90.0%), 7 of 11 (63.6%), and 10 of 12 (83.3%) participants had a positive antigen-specific plasma B cell response (defined as mean spot counts >10) 7 days after vaccination in the EFG-1, EFG-2, and placebo arms, respectively. The number of IgG-secreting plasma B cells across study arms before and after vaccination are shown in **Figure 5**.

**Figure 6:**
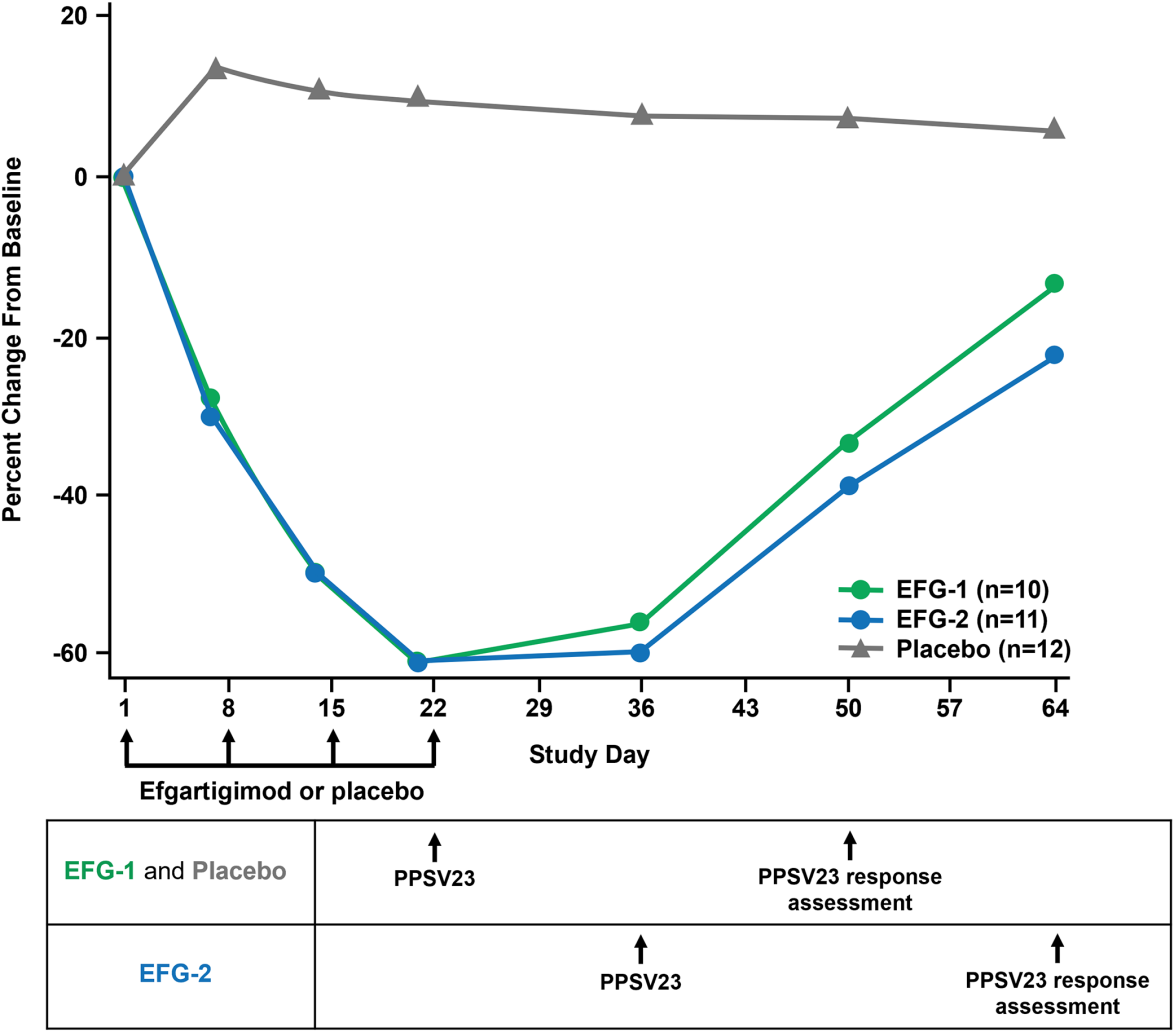
Mean percent change from baseline in IgG concentration over time by study arm. IgG was measured on Days 1, 7, 14, 21, 36, 50, and 64 in serum using validated methods. The timing of efgartigimod or placebo dosing, PPSV23 administration, and vaccine response assessment (at 4 weeks postvaccination) in each group is shown under the graph. EFG, efgartigimod; IgG, immunoglobulin G; PPSV23, 23-valent polyvalent pneumococcal polysaccharide vaccine.

The pattern of total IgG reduction over time was comparable between the EFG-1 and EFG-2 groups (**Figure 6**). Total IgG levels decreased by 61.4% from baseline (mean [SE], 994 [80] mg/dL) to Day 21 (mean [SE], 379 [28] mg/dL) in the EFG-1 group. In the EFG-2 group, total IgG levels decreased by 59.7% from baseline (mean [SE], 1141 [63] mg/dL) to Day 36 (mean [SE], 458 [28] mg/dL). Mean total IgG levels in the placebo group remained stable throughout the study (within a 13.5% margin of increase from baseline).

### 3.3 Safety assessments

A total of 84 TEAEs were reported for 10 participants (83.3%) in each arm (**Table 2**). No treatment-emergent serious AEs, grade ≥3 TEAEs, or deaths were reported. Two participants in the EFG-1 group experienced TEAEs that were deemed related to efgartigimod (1 participant with grade 1 erythema before receiving PPSV23 and 1 participant with grade 2 herpes zoster after vaccination). To minimize the spread of COVID-19, participants testing positive for SARS-CoV-2 at any point during the study were required to discontinue from the study intervention. Three participants discontinued the study due to COVID-19 infections (all grade 1), including 2 participants in the EFG-1 group and 1 participant in the EFG-2 group who completed treatment with efgartigimod but did not receive PPSV23. Treatment-emergent AEs occurring in ≥10% of patients in any group are summarized in **Table 2**. The most common TEAEs across the efgartigimod arms were headache, vomiting, fatigue, COVID-19 infection, abdominal discomfort, nausea, catheter site irritation, catheter site hematoma, and dizziness. During the study, catheter-site irritation (reported as irritation at cannula site) were reported in 0, 2 (16.7%), and 2 (16.7%) participants in EFG-1, EFG-2, and placebo, respectively. Catheter-site hematoma (reported as hematoma at cannula site and bruising at cannula site) were reported in 2 (16.7%), 0, and 1 (8.3%) participants in EFG-1, EFG-2, and placebo, respectively. All the events were severity grade 1 and nonserious. All the events were assessed as not related to efgartigimod by the investigator but related to the procedure.

**Table 2:** Summary of safety results (safety analysis set).

Event, n (%)	EFG-1 (n=12)	EFG-2 (n=12)	Placebo (n=12)
Any TEAEs	10 (83.3)	10 (83.3)	10 (83.3)
Related TEAEs	2 (16.7)	0 (0)	0 (0)
Grade ≥3 TEAEs	0 (0)	0 (0)	0 (0)
Treatment-emergent SAEs	0 (0)	0 (0)	0 (0)
TEAEs leading to treatment discontinuation	2 (16.7)	0 (0)^a^	0 (0)
Deaths	0 (0)	0 (0)	0 (0)
TEAEs reported in ≥10% of participants in any study arm, n (%)^b^			
Abdominal discomfort	1 (8.3)	2 (16.7)	2 (16.7)
Vomiting	0 (0)	4 (33.3)	1 (8.3)
Nausea	1 (8.3)	2 (16.7)	1 (8.3)
Fatigue	2 (16.7)	2 (16.7)	1 (8.3)
Catheter site irritation	0 (0)	2 (16.7)	2 (16.7)
Catheter site hematoma	2 (16.7)	0 (0)	1 (8.3)
Headache	4 (33.3)	4 (33.3)	1 (8.3)
Dizziness	0 (0)	2 (16.7)	1 (8.3)
Back pain	0 (0)	0 (0)	3 (25.0)
COVID-19	3 (25.0)	1 (8.3)	0 (0)

^a^ One participant in the EFG-2 group completed treatment with efgartigimod but did not receive PPSV23 before discontinuing the study due to COVID-19. ^b^Preferred Term according to the Medical Dictionary for Regulatory Activities Version 24.1. EFG, efgartigimod; PPSV23, 23-valent polyvalent pneumococcal polysaccharide vaccine; SAE, serious adverse event; TEAE, treatment-emergent adverse event.

## Discussion

The primary endpoint of this study was to evaluate the humoral response to PPSV23 by comparing pneumococcal capsular polysaccharide pre- and postvaccination IgG titers across study arms. Results from this exploratory study indicate that treatment with efgartigimod does not appear to impede the humoral immune response to PPSV23 in healthy volunteers. An increase in IgG levels after vaccination was observed for all 23 serotypes in both active treatment arms and the control arm, as indicated by the GMFR from baseline in pneumococcal capsular polysaccharide IgG titers. Although total IgG levels predictably decreased before vaccination in both efgartigimod groups, participants who received efgartigimod were still able to mount a humoral response to PPSV23, regardless of vaccination timing in relation to treatment with efgartigimod. These results are consistent with efgartigimod’s mechanism of action, whereby blocking the FcRn-mediated recycling pathway reduces total IgG levels but does not impact IgG antibody production or other parts of the immune response.

Consistent with pneumococcal capsular polysaccharide IgG titer results, a majority of participants in all groups exhibited a positive antigen-specific plasma B cell response 1 week after vaccination. Similar to the pneumococcal capsular polysaccharide IgG titers and previous literature (60), the opsonizing capacity of the selected anti–pneumococcal capsular polysaccharide antibodies 4 weeks postvaccination generally aligned with IgG seroresponses (although not assessed for correlation) but varied among the different serotypes and treatment arms in our study (57). This alignment supports the coherence between binding IgG titers and functional antibody activity across treatment arms. Nevertheless, efgartigimod treatment did not prevent an increase in functional antibody levels following PPSV23 vaccination, regardless of the timing of vaccination. Taken together, the results of this study support findings from previous studies demonstrating that treatment with efgartigimod did not impair the ability to mount efficient immune responses to vaccines (27, 35, 36, 46, 47).

Various factors aside from study treatment (efgartigimod or placebo) or vaccination timing may have contributed to the variability in immune parameters observed among groups in this study. Interindividual variation in immune response to different vaccines is well documented (60, 61); in a meta-analysis of studies evaluating the response to pneumococcal vaccine in healthy, immunocompetent subjects, the ratio of postvaccination-to-prevaccination titers varied widely for each serotype and among serotypes (range, 1.1-43.6) (60). In addition, despite randomization, differences in sex and age distribution among treatment arms due to the lack of stratification by sex and age may have contributed to differential immune responses in this study, complicating interpretation of immunogenicity outcomes and potentially affecting group comparability. Notably, a greater percentage of participants in the EFG-2 and placebo groups were male compared with the EFG-1 group. In a prior study evaluating antibody response to PPSV23 in healthy adults, female participants exhibited a significantly greater fold change in the sum of all serotype-specific IgG antibody levels compared with male participants (median, 4.26 vs 3.01, respectively; *P*=0.049); this difference was even greater among participants aged 50 years and older (62). A reduced immune response to pneumococcal vaccine in older patients has likewise been observed in other studies, including lesser increases in pneumococcal capsular polysaccharide IgG antibodies against some serotypes and reduced opsonophagocytic activity of postvaccination antibodies (63). As such, the older median age of participants randomized to placebo in the current study may have contributed to the lower percentages of participants with opsonizing titers above LLOQ and normal response criteria in this group. That said, a majority of participants in the placebo group met one of the individual criteria for normal response in the *post hoc* analyses (ie, >1.3 mg/L in ≥70% of serotypes). In contrast, differences in baseline pneumococcal serotype titers may have affected the potential for meeting the 2-fold increase threshold for normal response in the placebo group, in which baseline geometric mean titers were generally higher than in the efgartigimod groups for most serotypes. Prior pneumococcal exposure may have contributed to this finding, given the older age of participants in the placebo group; although our study intended to exclude individuals who had received a pneumococcal vaccine or experienced a pneumococcal infection within the prior 10 and 5 years, respectively, recall errors regarding previous infections or vaccinations are possible.

Another important factor to consider when interpreting the current findings includes the stringency of the definition used for normal response. A general limitation of studies evaluating response to PPSV23 is the lack of a firmly established threshold level for protection in the literature, making it difficult to determine a normal response to this type of vaccine. A stringent definition for normal response was used in this study (>1.3 mg/L against 70% of the serotypes tested, with at least a 2-fold increase in titers) (54). Additional *post hoc* analyses were performed on each of the individual criteria defining a normal response. The differences in immune response between the efgartigimod groups were less pronounced when considering the individual criteria that comprised the normal response definition. When considering only the criterion of a ≥2-fold increase in ≥70% of serotypes, the percentage of participants in the EFG-2 group exhibiting a response increased to nearly 73%, compared with 54.5% when considering both criteria. Furthermore, 1 participant in the EFG-2 group just missed the threshold for achieving both criteria in ≥70% of serotypes, meeting criterion for 15 of 23 serotypes (65.2%). If this participant had been included, the percentage of participants achieving normal response would have been 63.6% in the EFG-2 group, compared with 90.0% in the EFG-1 group. However, interpretation of the results of these *post hoc* analyses are limited by the small sample size. Additional factors that may have contributed to the different responses between the EFG-1 and EFG-2 arms include the normal variability in responses to PPSV23 and the aforementioned imbalance in sex distribution between the 2 groups.

Efgartigimod was well tolerated in a small sample of healthy participants in this study. The overall incidence of TEAEs was similar among the 2 efgartigimod arms and the placebo arm, and no serious or grade ≥3 TEAEs or deaths were observed. While COVID-19 infection accounted for all 3 study discontinuations (including 2 efgartigimod discontinuations), none of these infections were deemed related to efgartigimod treatment. Other than greater frequency of COVID-19 infections in the efgartigimod groups vs the placebo group (in which there were no such infections), the severity and types of TEAEs were consistent with previous studies of efgartigimod in both healthy participants and patients with IgG-mediated autoimmune disease (30, 33). Although this study showed a greater frequency of COVID-19 infections in the efgartigimod groups vs the placebo group, the relatively small sample size (n=12 per group in the safety analysis set) precludes the drawing of conclusions on whether there is an association between efgartigimod treatment and COVID-19 infection. As efgartigimod selectively reduces IgG levels, there exists a potential increased risk of infections; however, previous studies have shown that efgartigimod does not impact antibody production or levels of other immunoglobulins and does not substantially increase the risk of severe infections (27, 30, 32–36, 64). Additionally, previous placebo-controlled studies with larger sample sizes in participants with IgG-mediated autoimmune diseases have shown similar event rates of COVID-19 infection across participants receiving efgartigimod vs those receiving placebo. For example, in the ADVANCE study in participants with ITP, the event rate (number of events divided by patient years of follow-up) of COVID-19 was 0.18 in the efgartigimod IV group (n=86) compared with 0.16 in the placebo group (n=45) (65). In Stage B of the ADHERE study in participants with CIDP, the event rates of COVID-19 in the efgartigimod PH20 SC group (n=111) compared with the placebo group (n=110) were 0.39 and 0.40, respectively (65). These data are not suggestive of a large safety signal for COVID-19 with efgartigimod, but additional long-term data are required. Additional data are also required to assess the specific risk of efgartigimod treatment on viral reactivation, as this has only been investigated in 2 case report studies in 3 patients with MG (66, 67).

This was an exploratory study; no sample size calculations were performed, and no statistical hypotheses were tested. The relatively small sample size and variability of antibody response (among individual participants and serotypes) in this study may limit the reliability of subgroup comparisons, and the open-label study design may increase the risk of performance and detection bias. Additional studies in larger numbers of healthy volunteers or participants with IgG-mediated autoimmune diseases would be valuable. Another limitation was the short treatment duration, equivalent to a single efgartigimod cycle. Adults with gMG with anti–acetylcholine receptor antibodies, an approved indication for efgartigimod (37, 39, 68), often require more than 1 treatment cycle. However, prior studies investigating humoral immune responses to various T cell–dependent and –independent vaccines in participants with gMG and pemphigus incorporated the equivalent of multiple treatment cycles (27, 35, 69), and additional longer-term studies that incorporate assessment of vaccine immune responses are ongoing (46). It is also unknown whether any patients developed pneumococcal infections after this study was concluded. Longer-term observational studies in efgartigimod-treated patients with autoantibody-mediated diseases who receive PPSV23 would be helpful in ascertaining whether the humoral responses observed in this study correlate with protection from clinical infection, as well as providing additional data to evaluate long-term immunogenicity and durability of the vaccine response. Additionally, as this study was conducted in healthy human volunteers, results may not be generalizable to patients with IgG-mediated autoimmune disease who may have altered immune function and response to vaccines and receive concomitant immunosuppressive therapy (70). Additional studies confirming the findings of the current study in those receiving other types of vaccines during treatment with efgartigimod, including live and live-attenuated vaccines, are warranted.

In conclusion, administration of efgartigimod in healthy individuals had no apparent effect on the generation of T cell–independent humoral immune responses to the PPSV23 vaccine, regardless of timing of vaccination relative to efgartigimod treatment. Antibody and antigen-specific plasma B cell responses were observed among all groups in the study, and an opsonophagocytic assay that reproduces the protective mechanism of these antibodies (57, 58) indicated that they were functional. Importantly, participants were able to mount these responses even when the vaccination was given at the time of maximal reduction in total IgG. The reduction in total IgG was similar to previous studies, in which reductions in total IgG levels were shown to be incomplete and transient, returning to baseline levels between study Days 36 to 64 (30, 33). The results are consistent with several previous animal and human clinical studies of efgartigimod that showed a preserved immune response to vaccines (27, 35, 36, 46, 47). Taken together, all these studies provide support for administering recommended nonlive vaccines at any time during the efgartigimod treatment cycle (68), allowing for flexibility and necessary protection. Further observational studies on vaccine response in efgartigimod-treated participants with IgG-mediated autoimmune diseases are ongoing.

## Data Availability

The datasets presented in this article are not readily available, however argenx is committed to responsible data sharing regarding the studies they fund. These data can be requested by qualified researchers who engage in rigorous independent scientific research and will only be provided after review and approval of a research proposal and statistical analysis plan and execution of a data-sharing agreement. Data requests can be submitted at any time, and the data will be accessible for 12 months. Requests to access the datasets should be directed to ESR@argenx.com.
