# Supplementary material for: Humoral immune response to polyvalent pneumococcal vaccine in healthy participants receiving efgartigimod: a randomized, open-label, placebo-controlled, parallel-group phase 1 trial
Source: Front Immunol. 2026 May 7;17:1799480. doi: 10.3389/fimmu.2026.1799480 (PMC13191902; doi:10.3389/fimmu.2026.1799480)
Supplement: Supplementary file 1 [file DataSheet1.docx]

Supplementary Material
For the original article: Humoral Immune Response to Polyvalent Pneumococcal Vaccine in Healthy Participants Receiving Efgartigimod:
 A Randomized, Open-Label, Placebo-Controlled,
Parallel-Group Phase 1 Trial

# Supplementary Tables

**Supplementary Table S1.** Upper Limit of Detection for Each Serotype Using the Multi-Analyte Immunodetection Method. Each upper limit threshold corresponded to the highest standard used for that serotype.

| **Serotype number** | **Upper limit threshold (µg/mL)** |
| --- | --- |
| 1 | 111 |
| 2 | 114 |
| 3 | 44 |
| 4 | 28 |
| 5 | 154 |
| 6B | 101 |
| 7F | 110 |
| 8 | 86 |
| 9N | 41 |
| 9V | 61 |
| 10A | 77 |
| 11A | 39 |
| 12F | 30 |
| 14 | 222 |
| 15B | 81 |
| 17F | 67 |
| 18C | 72 |
| 19A | 141 |
| 19F | 139 |
| 20 | 54 |
| 22F | 72 |
| 23F | 53 |
| 33F | 47 |

**Supplementary Table S2.** Geometric Mean and Coefficient of Variation of Absolute Pneumococcal Capsular Polysaccharide Titers on Day 1 and 4 Weeks Postvaccination.

| **Serotype** | **EFG-1 (n=10)** | | | | **EFG-2 (n=11)** | | | | **Placebo (n=12)** | | | |
| --- | --- | --- | --- | --- | --- | --- | --- | --- | --- | --- | --- | --- |
|  | Day 1 (baseline)^a^ | | 4 weeks postvaccination^b^ | | Day 1 (baseline)^a^ | | 4 weeks postvaccination^b^ | | Day 1 (baseline)^a^ | | 4 weeks postvaccination^b^ | |
|  | Geometric mean, CV (%) | 95 % CI | Geometric mean, CV (%) | 95 % CI | Geometric mean, CV (%) | 95 % CI | Geometric mean, CV (%) | 95 % CI | Geometric mean, CV (%) | 95 % CI | Geometric mean, CV (%) | 95 % CI |
| 1 | 0.3, 94.3 | 0.2, 0.5 | 4.6, 85.6 | 2.2, 9.5 | 0.6, 293.4 | 0.2, 1.8 | 4.2, 167.3 | 1.1, 16.2 | 0.7, 86.4 | 0.3, 1.4 | 8.2, 153.3 | 3.3, 20.4 |
| 2 | 0.4, 97.5 | 0.2, 0.7 | 3.8, 200.4 | 1.6, 9.2 | 0.4, 106.5 | 0.2, 0.8 | 2.9, 135.6 | 1.4, 6.3 | 0.6, 154.2 | 0.3, 1.3 | 6.4, 150.3 | 2.4, 16.8 |
| 3 | 0.5, 143.1 | 0.2, 1.1 | 2.3, 138.8 | 0.9, 5.7 | 0.8, 170.5 | 0.3, 1.9 | 2.3, 165.3 | 0.8, 6.3 | 0.4, 104.5 | 0.2, 0.8 | 1.7, 118.2 | 0.7, 3.9 |
| 4 | 0.2, 29.3 | 0.2, 0.3 | 2.0, 98.9 | 1.0, 4.3 | 0.3, 96.2 | 0.2, 0.4 | 0.9, 106.0 | 0.4, 2.0 | 0.4, 175.6 | 0.2, 0.8 | 0.7, 151.8 | 0.3, 1.8 |
| 5 | 0.5, 110.4 | 0.2, 1.1 | 3.7, 117.7 | 1.3, 10.8 | 0.7, 175.7 | 0.3, 1.9 | 4.5, 166.7 | 1.0, 20.7 | 1.6, 280.3 | 0.5, 5.0 | 12.7, 112.9 | 3.1, 52.9 |
| 6B | 0.4, 193.7 | 0.2, 0.7 | 2.7, 73.2 | 1.2, 5.9 | 0.4, 90.6 | 0.2, 0.8 | 1.8, 115.8 | 0.6, 5.2 | 0.7, 110.0 | 0.3, 1.4 | 3.1, 195.8 | 1.4, 7.1 |
| 7F | 1.5, 123.2 | 0.6, 4.1 | 5.4, 121.2 | 1.4, 21.2 | 0.5, 106.1 | 0.3, 0.9 | 3.5, 169.9 | 1.0, 11.8 | 1.0, 316.7 | 0.3, 2.9 | 6.8, 161.8 | 2.2, 20.6 |
| 8 | 0.5, 120.4 | 0.2, 1.1 | 8.7, 88.2 | 2.5, 30.3 | 0.4, 78.1 | 0.2, 0.6 | 4.4, 131.0 | 1.5, 12.6 | 0.5, 187.2 | 0.2, 1.2 | 3.8, 74.2 | 2.2, 6.7 |
| 9N | 0.5, 111.3 | 0.2, 1.2 | 2.4, 181.3 | 0.8, 7.1 | 0.6, 108.6 | 0.3, 1.2 | 4.3, 136.6 | 1.6, 12.0 | 0.4, 188.8 | 0.2, 0.8 | 3.2, 141.4 | 1.1, 9.0 |
| 9V | 0.6, 110.2 | 0.3, 1.2 | 2.7, 163.2 | 0.8, 8.5 | 0.5, 155.6 | 0.2, 1.0 | 1.6, 167.6 | 0.5, 4.5 | 0.5, 140.1 | 0.2, 0.9 | 1.9, 95.3 | 0.9, 3.9 |
| 10A | 0.3, 122.1 | 0.2, 0.5 | 2.4, 174.3 | 0.8, 7.1 | 0.6, 159.8 | 0.3, 1.1 | 3.0, 172.9 | 0.9, 10.0 | 0.6, 100.5 | 0.3, 1.3 | 5.9, 79.8 | 2.7, 12.9 |
| 11A | 0.9, 179.1 | 0.3, 2.2 | 3.0, 93.1 | 1.2, 7.3 | 0.5, 279.2 | 0.2, 1.3 | 1.5, 228.2 | 0.5, 3.9 | 1.1, 126.6 | 0.5, 2.5 | 4.4, 88.3 | 2.4, 8.1 |
| 12F | 0.2, 15.1 | 0.2, 0.2 | 2.0, 140.5 | 0.5, 7.8 | 0.3, 197.7 | 0.2, 0.7 | 1.4, 173.3 | 0.5, 4.3 | 0.5, 290.3 | 0.2, 1.3 | 2.2, 152.8 | 0.8, 5.7 |
| 14 | 1.3, 96.8 | 0.5, 3.3 | 7.8, 198.1 | 1.9, 32.2 | 0.8, 111.9 | 0.3, 2.0 | 4.4, 149.6 | 1.0, 20.1 | 1.4, 183.4 | 0.4, 4.8 | 8.1, 117.4 | 2.5, 26.5 |
| 15B | 0.7, 152.8 | 0.2, 1.8 | 4.3, 112.4 | 1.2, 15.7 | 0.6, 139.2 | 0.3, 1.4 | 3.0, 156.4 | 0.8, 10.6 | 1.4, 180.3 | 0.5, 3.5 | 8.0, 96.7 | 3.5, 18.5 |
| 17F | 0.8, 81.2 | 0.4, 1.7 | 9.0, 99.8 | 2.5, 32.0 | 0.4, 131.4 | 0.2, 0.9 | 2.2, 195.5 | 0.7, 7.6 | 1.4, 149.0 | 0.4, 4.3 | 9.3, 116.1 | 4.6, 18.8 |
| 18C | 0.9, 111.1 | 0.4, 1.8 | 4.6, 98.1 | 1.6, 12.8 | 0.8, 99.4 | 0.3, 1.8 | 2.5, 170.9 | 0.7, 9.1 | 1.6, 264.6 | 0.6, 4.4 | 9.9, 123.0 | 4.9, 20.1 |
| 19A | 1.1, 199.0 | 0.5, 2.4 | 2.4, 90.2 | 0.9, 6.2 | 0.8, 129.2 | 0.4, 1.7 | 4.1, 85.7 | 1.5, 11.1 | 1.6, 294.0 | 0.5, 5.2 | 3.5, 244.7 | 1.1, 10.8 |
| 19F | 1.8, 71.7 | 0.9, 3.7 | 7.1, 141.9 | 2.3, 22.1 | 1.7, 206.9 | 0.8, 3.6 | 5.4, 106.4 | 2.1, 13.6 | 2.1, 302.7 | 0.8, 5.3 | 8.2, 195.8 | 3.8, 17.6 |
| 20 | 1.3, 119.5 | 0.6, 2.8 | 4.7, 107.2 | 2.0, 11.0 | 1.5, 118.2 | 0.7, 3.1 | 3.8, 161.0 | 1.5, 9.5 | 2.4, 157.7 | 1.0, 5.6 | 7.3, 114.8 | 2.8, 19.4 |
| 22F | 0.2, 43.2 | 0.2, 0.3 | 2.8, 147.4 | 1.1, 7.2 | 0.4, 212.9 | 0.2, 1.0 | 2.8, 196.0 | 0.9, 9.1 | 0.3, 329.6 | 0.1, 0.9 | 1.6, 253.1 | 0.5, 4.8 |
| 23F | 0.5, 120.2 | 0.2, 1.1 | 4.9, 117.8 | 1.7, 14.2 | 0.5, 175.7 | 0.2, 1.3 | 1.8, 202.7 | 0.5, 5.7 | 0.5, 268.5 | 0.2, 1.1 | 1.9, 130.7 | 0.7, 4.9 |
| 33F | 0.5, 182.6 | 0.2, 1.1 | 4.1, 141.7 | 1.3, 12.5 | 0.5, 72.8 | 0.3, 0.7 | 3.2, 167.2 | 1.3, 7.8 | 1.5, 197.5 | 0.6, 3.8 | 8.2, 92.1 | 2.5, 27.0 |

^a^Baseline is defined as the last observation recorded before the first study drug administration. ^b^Participants in the EFG-1 and placebo groups received PPSV23 on Day 22; the data shown for these groups are from Day 50. Participants in the EFG-2 group received PPSV23 on Day 36; the data shown for this group are from Day 64. Abbreviations: CV, coefficient of variation; EFG, efgartigimod; PPSV23, 23-valent polyvalent pneumococcal polysaccharide vaccine.

**Supplementary Table S3.** Summary of Participants Achieving Normal Response and Individual Response Criteria per Serotype and for Serotypes Combined.

| **Serotype** | **EFG-1 (n=10)** | | | **EFG-2 (n=11)** | | | **Placebo (n=12)** | | |
| --- | --- | --- | --- | --- | --- | --- | --- | --- | --- |
|  | **≥2-fold increase, n (%)** | **>1.3 mg/L,**  **n (%)** | **Both,**  **n (%)^a^** | **≥2-fold increase, n (%)** | **>1.3 mg/L,**  **n (%)** | **Both,**  **n (%)^a^** | **≥2-fold increase, n (%)** | **>1.3 mg/L,**  **n (%)** | **Both,**  **n (%)^a^** |
| 1 | 10 (100.0) | 9 (90.0) | 9 (90.0) | 11 (100.0) | 6 (54.5) | 6 (54.5) | 11 (91.7) | 10 (83.3) | 9 (75.0) |
| 2 | 10 (100.0) | 8 (80.0) | 8 (80.0) | 11 (100.0) | 10 (90.9) | 10 (90.9) | 11 (91.7) | 11 (91.7) | 10 (83.3) |
| 3 | 10 (100.0) | 6 (60.0) | 6 (60.0) | 8 (72.7) | 7 (63.6) | 7 (63.6) | 9 (75.0) | 7 (58.3) | 7 (58.3) |
| 4 | 9 (90.0) | 9 (90.0) | 9 (90.0) | 9 (81.8) | 5 (45.5) | 5 (45.5) | 4 (33.3) | 4 (33.3) | 3 (25.0) |
| 5 | 9 (90.0) | 7 (70.0) | 7 (70.0) | 10 (90.9) | 7 (63.6) | 7 (63.6) | 10 (83.3) | 9 (75.0) | 8 (66.7) |
| 6B | 9 (90.0) | 8 (80.0) | 8 (80.0) | 9 (81.8) | 6 (54.5) | 6 (54.5) | 9 (75.0) | 10 (83.3) | 7 (58.3) |
| 7F | 8 (80.0) | 7 (70.0) | 7 (70.0) | 10 (90.9) | 6 (54.5) | 6 (54.5) | 9 (75.0) | 10 (83.3) | 8 (66.7) |
| 8 | 9 (90.0) | 9 (90.0) | 9 (90.0) | 10 (90.9) | 9 (81.8) | 9 (81.8) | 10 (83.3) | 10 (83.3) | 8 (66.7) |
| 9N | 9 (90.0) | 7 (70.0) | 7 (70.0) | 11 (100.0) | 9 (81.8) | 9 (81.8) | 10 (83.3) | 8 (66.7) | 7 (58.3) |
| 9V | 8 (80.0) | 8 (80.0) | 8 (80.0) | 9 (81.8) | 5 (45.5) | 5 (45.5) | 8 (66.7) | 7 (58.3) | 5 (41.7) |
| 10A | 9 (90.0) | 7 (70.0) | 7 (70.0) | 9 (81.8) | 8 (72.7) | 8 (72.7) | 11 (91.7) | 10 (83.3) | 9 (75.0) |
| 11A | 9 (90.0) | 9 (90.0) | 9 (90.0) | 9 (81.8) | 6 (54.5) | 6 (54.5) | 10 (83.3) | 11 (91.7) | 10 (83.3) |
| 12F | 7 (70.0) | 5 (60.0) | 6 (60.0) | 8 (72.7) | 6 (54.5) | 6 (54.5) | 9 (75.0) | 8 (66.7) | 7 (58.3) |
| 14 | 9 (90.0) | 9 (90.0) | 9 (90.0) | 8 (72.7) | 7 (63.6) | 6 (54.5) | 9 (75.0) | 10 (83.3) | 8 (66.7) |
| 15B | 7 (70.0) | 8 (80.0) | 7 (70.0) | 9 (81.8) | 7 (63.6) | 7 (63.6) | 8 (66.7) | 10 (83.3) | 8 (66.7) |
| 17F | 9 (90.0) | 8 (80.0) | 8 (80.0) | 10 (90.9) | 6 (54.5) | 6 (54.5) | 11 (91.7) | 12 (100.0) | 11 (91.7) |
| 18C | 9 (90.0) | 8 (80.0) | 8 (80.0) | 10 (90.9) | 6 (54.5) | 6 (54.5) | 10 (83.3) | 12 (100.0) | 10 (83.3) |
| 19A | 7 (70.0) | 7 (70.0) | 6 (60.0) | 9 (81.8) | 9 (81.8) | 8 (72.7) | 4 (33.3) | 9 (75.0) | 4 (33.3) |
| 19F | 9 (90.0) | 9 (90.0) | 9 (90.0) | 10 (90.9) | 9 (81.8) | 9 (81.8) | 10 (83.3) | 12 (100.0) | 10 (83.3) |
| 20 | 9 (90.0) | 9 (90.0) | 9 (90.0) | 11 (100.0) | 9 (81.8) | 9 (81.8) | 7 (58.3) | 11 (91.7) | 6 (50.0) |
| 22F | 9 (90.0) | 8 (80.0) | 8 (80.0) | 10 (90.9) | 5 (45.5) | 5 (45.5) | 8 (66.7) | 7 (58.3) | 6 (50.0) |
| 23F | 9 (90.0) | 9 (90.0) | 9 (90.0) | 9 (81.8) | 5 (45.5) | 5 (45.5) | 7 (58.3) | 6 (50.0) | 5 (41.7) |
| 33F | 9 (90.0) | 9 (90.0) | 9 (90.0) | 11 (100.0) | 9 (81.8) | 9 (81.8) | 9 (75.0) | 9 (75.0) | 7 (58.3) |
| **Combined (≥70%)** | **9 (90.0)** | **9 (90.0)** | **9 (90.0)** | **8 (72.7)** | **6 (54.5)** | **6 (54.5)** | **9 (75.0)** | **10 (83.3)** | **4 (33.3)** |

^a^Encompasses participants who simultaneously achieved a ≥2-fold increase and absolute titer >1.3 mg/L for each serotype and for combined (≥70%) serotypes (last row) at the respective assessment time points (Week 50 for EFG-1 and placebo and Week 64 for EFG-2). Abbreviations: EFG, efgartigimod.

# Supplementary Figures

**
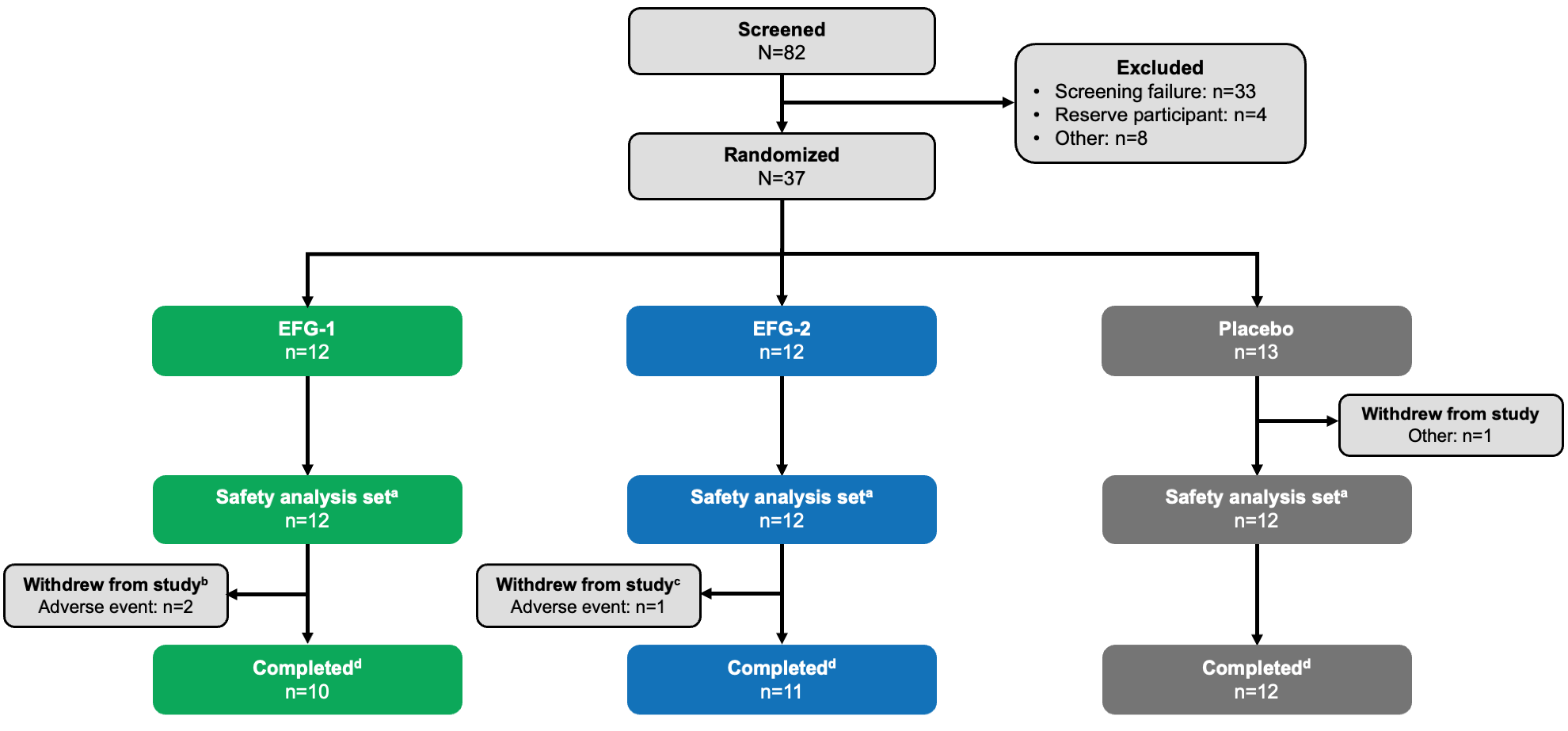
**

**Supplementary Figure S1.** Participant Disposition Diagram. ^a^The safety analysis set consisted of all randomized participants who received ≥1 dose of investigational medical product. ^b^Two participants in the EFG-1 group discontinued the study due to COVID-19 infection.
^c^An additional participant in the EFG-2 group completed treatment with efgartigimod but discontinued due to COVID-19 infection before receiving PPSV23. ^d^Participants completing the study encompassed the PD analysis set for each group. The PD analysis set consisted of participants from the safety analysis set who received PPSV23. Abbreviations: EFG, efgartigimod; PD, pharmacodynamic; PPSV23, 23-valent polyvalent pneumococcal polysaccharide vaccine.


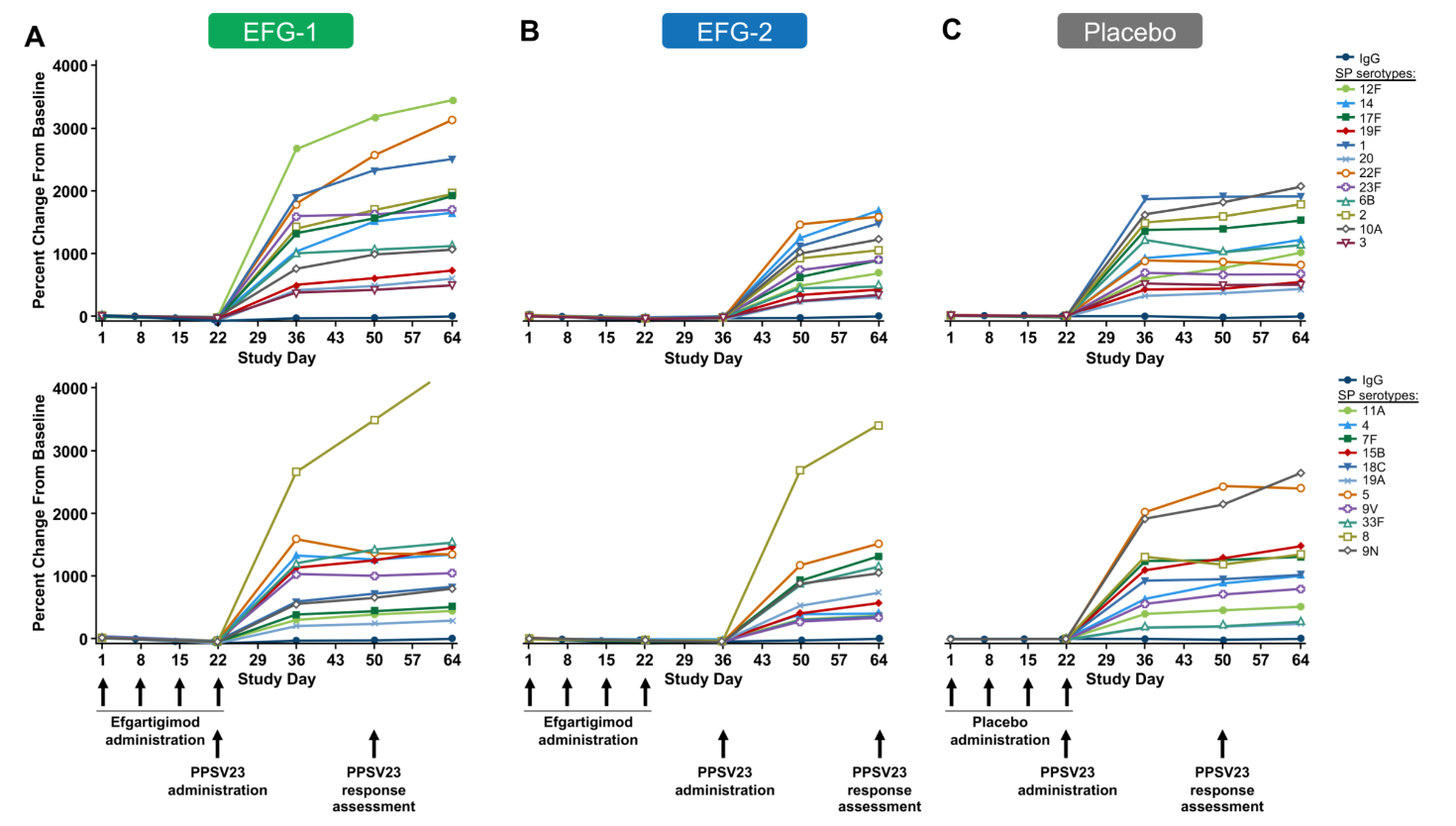


**Supplementary Figure S2.** Mean Percent Change From Prevaccination in Total IgG and Pneumococcal Capsular Polysaccharide Titers in the EFG-1 (n=10; panel A), EFG-2 (n=11; panel B), and Placebo (n=12; panel C) Groups. Mean percent changes for all *Streptococcus pneumoniae* serotypes contained within PPSV23 are distributed across the top and bottom graphs in each panel for legibility. The timing of efgartigimod or placebo dosing, PPSV23 administration, and vaccine response assessment (at 4 weeks postvaccination) for each respective group is shown under the bottom graph in each panel. Abbreviations: EFG, efgartigimod; IgG, immunoglobulin G; PPSV23, 23-valent polyvalent pneumococcal polysaccharide vaccine; SP, *Streptococcus pneumoniae.*

**
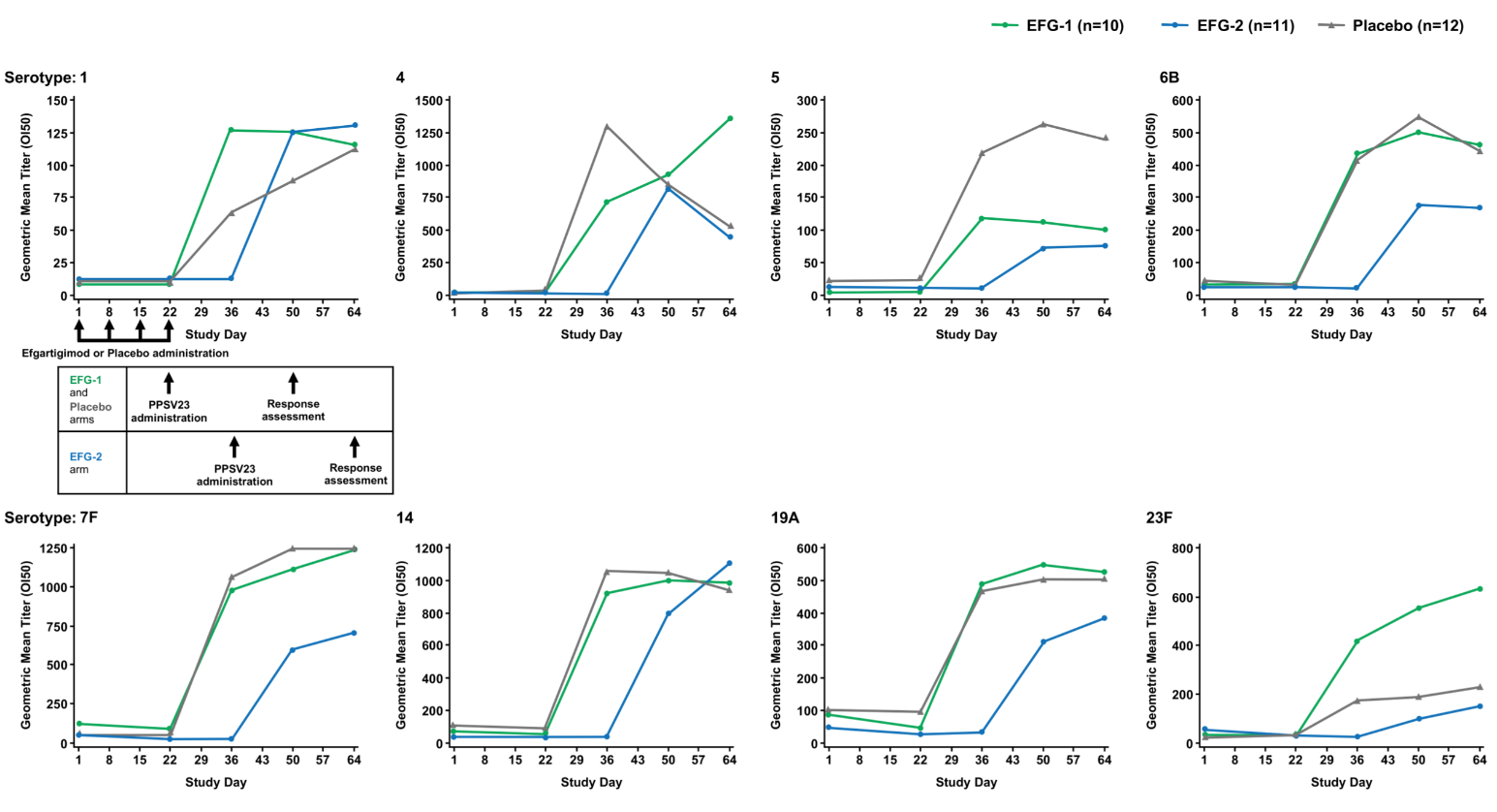
**

**Supplementary Figure S3.** Geometric Mean Plot of Functional Antibody Titers as Measured by Opsonophagocytic Assay Over Time for the 8 Pneumococcal Serotypes Assessed. The timing of efgartigimod or placebo dosing, PPSV23 administration, and vaccine response assessment (at 4 weeks postvaccination) in each group is shown under the top left graph. Abbreviations: EFG, efgartigimod; OI50, opsonization index 50%; PPSV23, 23-valent polyvalent pneumococcal polysaccharide vaccine.
